# Proactive strategies for an inclusive faculty search process

**DOI:** 10.1038/s42003-022-03545-z

**Published:** 2022-06-16

**Authors:** Karena H. Nguyen, Kyle Thomas, Robert C. Liu, Anita H. Corbett

**Affiliations:** 1 Department of Biology, Emory University, Atlanta, GA USA; 2 Department of Biomedical Engineering, Emory University, Atlanta, GA USA; 3 Silvio O. Conte Center for Oxytocin and Social Cognition and Center for Translational Social Neuroscience, Emory University, Atlanta, GA USA

**Keywords:** Institutions, Careers

## Abstract

A case study on an inclusive faculty hiring process at Emory University provides advice for institutions creating their own hiring panels and workflows.

## Teaser

Each faculty search for new academic colleagues consumes significant time and energy for all parties involved, from the applicants to the search committee, other engaged colleagues, and leadership that aid in negotiations along the way. The goal here is to present specific approaches employed in a recent search process that were designed to contribute to an inclusive search process. Challenges and opportunities presented by performing the search during a global pandemic are incorporated. The detailed description of each step in the search should both be helpful to those planning their own faculty searches and to applicants who will benefit from seeing the process demystified.

During the academic year 2020–2021, the Biology Department at Emory University conducted a faculty search that was designed to continue the success of the department in building an inclusive department^1,2^. Several actions were taken to ensure an equitable search process despite the challenges and biases inherent in search processes^3–7^ that had the potential to be further exacerbated by a raging global pandemic^8^.

The search was approved for two tenure track faculty positions with no formal limitation on the academic rank of the hires. The entire department invested in efforts to engage in a very broad search that would have the highest likelihood of identifying and recruiting outstanding researchers with a commitment to teaching and who also showed evidence of actions toward the mission of diversity, equity, and inclusion. These values reflect the Biology Department status within Emory College of Arts and Sciences, but also place front and center the need to establish an outstanding, extramurally funded research program consistent with success at an R1 institute such as Emory University.

**Fig. 1:**
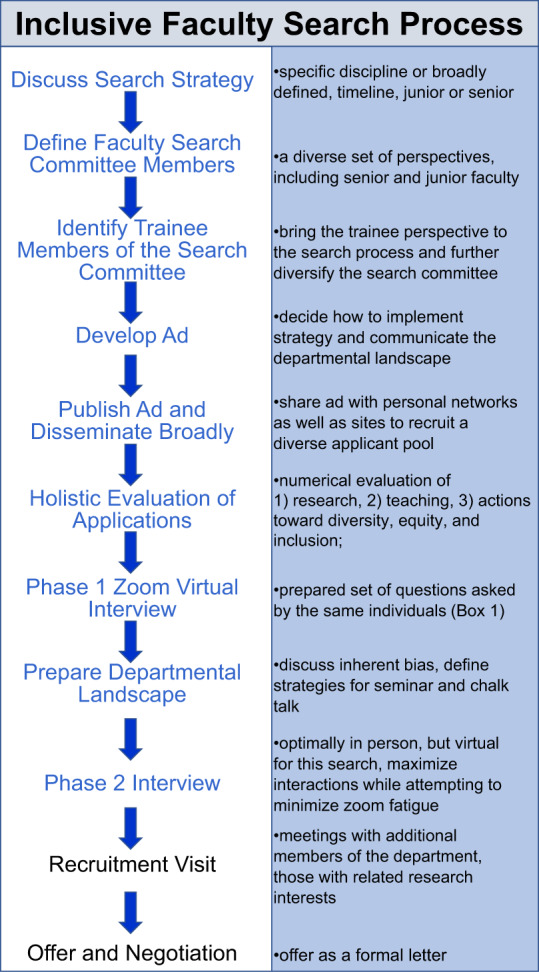
An overview of the faculty search process. All steps depicted in blue were modified to some extent to achieve the goal of having an inclusive faculty search process. Each of the steps modified in this search is addressed in detail in the text.

For this search, we modified aspects of our traditional search in several ways that included the strategy for the search, the ad that described the position, the composition of the search committee, and aspects of the process (performed largely virtually due to COVID-19). We were fortunate to have strong support from leadership at all levels from the Department to the Dean and Dean’s Office to allow us to make changes aligned with the goals of the search. A general overview of the search process is shown in Fig. 1, with details about each step expanded upon in the text.

## The strategy: a broad search

Traditionally, the Biology Department in Emory College of Arts and Science, which shares a physical location with the School of Medicine at Emory University, has performed faculty searches designed to build in three scientific focus areas within the department. However, with the goal of attracting a deep pool of highly qualified applicants, we decided to make this search as broad as possible and advertised for a “biologist”, clearly stating in the position advertisement, “This search is open for any area of biology”. Such an approach, we reasoned, would allow the most candidates to apply and consider themselves qualified for this position. This approach would counter the issue that women and those from historically excluded and underrepresented groups do not apply for positions unless they meet all the criteria listed^9,10^ and might also increase interest in the position from scientists typically less represented in the applicant pool^11^.

### The ad: highlight the inclusive nature of the department

Beyond a general strategy of spreading a wide net to attract a deep pool of outstanding applicants, members of the department put time and effort into modifying the typical template for the position advertisement. The requested material was fairly standard consisting of a cover letter, CV, research statement, teaching statement, and diversity statement (with the statements limited to 6 pages total), and three letters of recommendation. However, we wanted to use the ad to achieve a goal beyond soliciting materials; specifically, we sought to showcase the values within the department that we were seeking in the candidates we wished to recruit. To this end, a link was included to the department ‘statement supporting inclusivity in our community’, which is posted on the department homepage. We also included the following statement to emphasize how this search fit within the context of our long-term goals for the department, “This search is part of a multi-year Emory initiative to foster equity and to promote the success of our diverse student population.” Finally, modeled on other ads that made this point, we encouraged applicants who might not yet have that final paper in press to still send their application by stating, “We encourage outstanding candidates who are early in their post-doctoral research training to apply, even if their primary findings are not yet published.” This ad was placed with *Chronicle of Higher Education*, the SACNAS (Society for Advancement of Chicanos/Hispanics & Native Americans in Science) website, and *Science*. However, all members of the faculty reached out to their networks to solicit applicants. The ad was also shared on Twitter. Overall, the strategy of a broad search was successful as we received 585 completed applications for the position. Of these applicants, 60% were male, 35% were female, 3% declined to identify as male or female and <1% of applicants selected Other. Within this pool, 13% of applicants self-identified as belonging to a group underrepresented in science, technology, engineering, and mathematics (STEM) fields, which includes Hispanic/Latino (any race), American Indian/Alaskan Native, Black/African American, and Native Hawaiian/Pacific Islander. Finally, 4% of applicants self-identified as having or having had a disability while 5% declined to report any disability status.

### The search committee: broad representation including trainees

The search committee consisted of six faculty members, including, per Emory rules, one faculty member from outside the department. In addition to these six faculty members, the Department Chair routinely serves as an ex officio member of the committee to answer any questions that arise. We strive to represent diverse research areas in the broad department and also include faculty at different career stages^12^. In addition, for the first time, we included two trainees as formal members of the search committee. While we have previously had trainees meet with candidates and we have solicited their feedback via a survey, this was an informal process and few, if any, of the same trainees would meet with different candidates. To formalize the inclusion of trainees on the search committee, all department graduate students and post-docs were sent an e-mail inviting them to submit a CV and a short statement about their interest in serving on the search committee. One graduate student and one-post-doctoral fellow were then selected to serve on the committee, with the criteria that neither trainee was from the research group of a faculty member serving on the committee and they also were not from the same research group. This strategy ensured that broad perspectives from the department (and beyond the department) were represented on the search committee. A challenge in including trainees in the relatively small search committee was to consider how the trainees would be integrated into the process to ensure that their voices were valued while taking into consideration their somewhat limited experience in evaluating faculty candidates. Below, we describe how trainees participated in the process, and also encourage departments to discuss and adapt how they may do the same for future searches^13^. Beyond forming the search committee, Emory University mandates implicit bias training for all search committee members, which included the trainees. This training for the search committee is supplemented by additional discussion to prepare the department more broadly for the search, which is discussed below. This search did not include a Diversity Advisor/Advocate on the search committee, which is a strategy being implemented for many searches^14^, but rather embraced the commitment of the search committee members and broader department to building an inclusive and diverse community. Prior to the search, the search committee chair did meet with the Chief Diversity Officer of the University together with several other search committee members. In the future, this meeting should include the entire search committee, including trainees.

### Evaluating applications: a holistic process

**Fig. 2:**
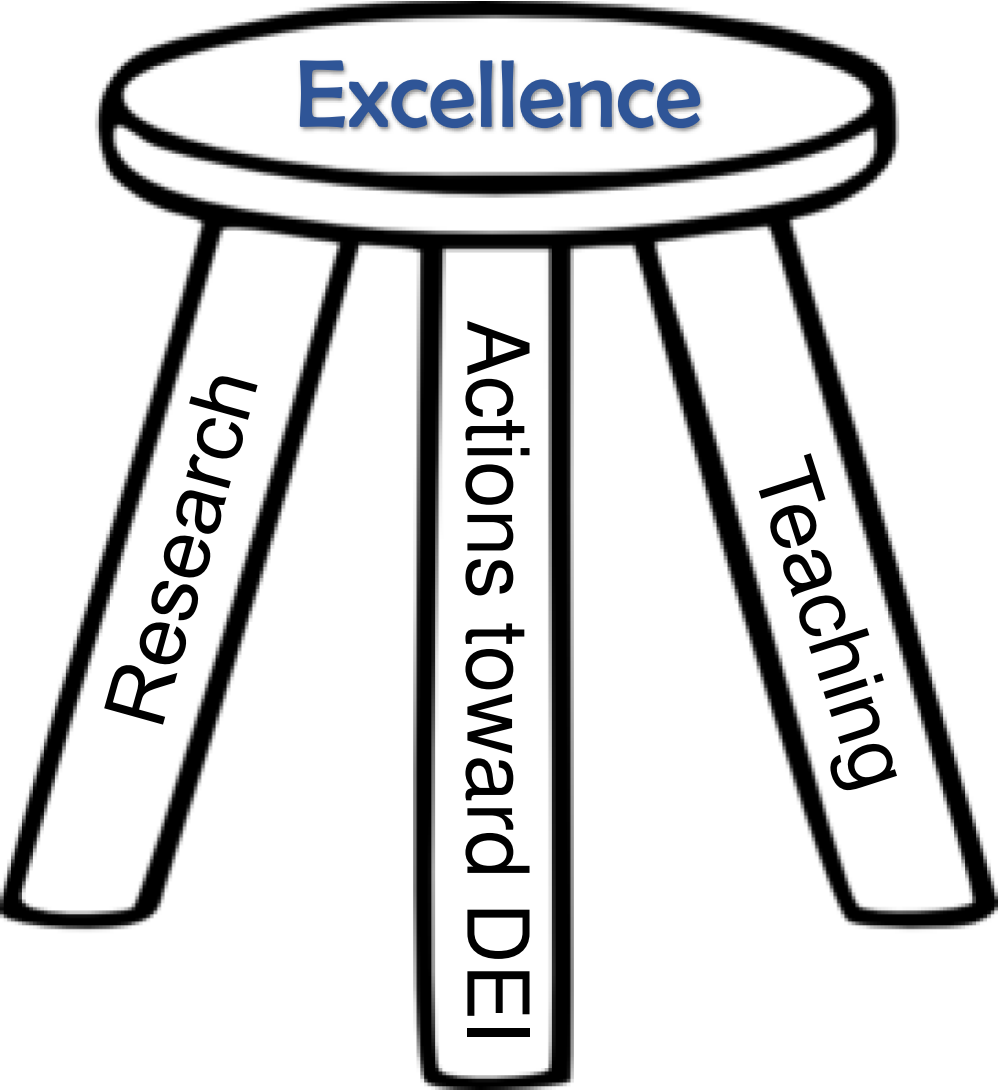
Evaluation of applicants used a three-pronged approach. We equally valued Research, Teaching, and Actions toward Diversity, Equity, and Inclusion (DEI) as supporting the Excellence we were seeking in candidates. We scored each of these categories in our initial evaluation of 585 completed applications consisting of a cover letter (CV), research, teaching, and DEI statements as well as recommendation letters.

With the success of soliciting 585 applications came the burden of adequately and equitably evaluating this large number of applications. We decided this task would fall to faculty on the search committee and trainees would be integrated into the search at subsequent stages. In part, this decision was made simply to avoid burdening trainees with a very time-consuming task. With our goal of contributing to an inclusive departmental culture at the forefront, we opted to equally value: research, teaching, and actions toward diversity, equity, and inclusion (Fig. 2). For each of these categories, we scored on a scale of 1–5 according to the following scale: 1: outstanding; 2: great; 3: average compared to group of applicants as a whole; 4: below average; 5: not acceptable. We then divided the six faculty members on the committee into two groups of three reviewers, with the members of each group having distinct scientific expertize, and randomly assigned each applicant file to two reviewers, with one from each group of three. Faculty reviewers were tasked with reading each file in the following order: (1) cover letter, (2) diversity statement, (3) teaching statement, (4) research statement, (5) CV, (6) letters of recommendation. This strategy ensured that all aspects of the files were evaluated using the same process.

Evaluating each of the categories was challenging, particularly for such a large number of candidates. No specific criteria were defined for these evaluations in part because the diverse scientific areas would have made direct comparison challenging. Both actions toward diversity, equity, and inclusion and teaching could be evaluated by experience described in the applicants’ statements as well as details on their CVs. In addition, the trainee members of the search committee updated a rubric to evaluate DEI statements^15^ (Supplementary Note 1). Most applicants focus on the research statement, so the mere act of taking the teaching and DEI aspects of the application seriously shows some levels of commitment. Evaluating research potential is quite challenging^16^ and, with so many candidates, even evaluating the impact of previous accomplishments was very difficult. This was made particularly challenging as we explicitly stated in the ad that applicants who have not yet published their major work from their post-doctoral training were encouraged to apply. Ultimately, productivity was evaluated based on publications, although this latter point was taken into consideration. For example, manuscripts posted to *bioRxiv* were considered^17,18^, as well as data presented in the research statement. A challenge in this approach is documented biases in the publishing process^19^. Another key element was some evidence of ability to obtain extramural funding^20–22^. This could be a pre/post-doctoral fellowship, an NIH K99, or any other form of extramural support, taking into consideration that not all applicants have the same ability to apply for funding at different stages of training or in particular fields that are historically awarded less funding. Finally, the research statement was critically important for communicating both the impact of past accomplishments and a clear plan for future studies. A point of consideration is always clarity about how the research path will diverge from that of previous mentors.

Following the numerical evaluation of all 585 applications, nearly 200 applications for each member of the search committee, the three category scores (research, teaching, and actions toward diversity, equity, and inclusion) were totaled and averaged to identify a top slate of applicants for further consideration. As a complement to this quantitative approach, each reviewer was asked to select ~10 applicants for further consideration, using the same review criteria, but potentially weighing aspects of the application differently. Following this process, the group of top candidates (~45 total) was further narrowed with respect to research by reaching out to colleagues in the department or beyond familiar with the research areas. These colleagues were charged with evaluating the impact of the previous research and the potential of the planned research. Although a number of candidates had not yet published their major findings from their post-doctoral work, some applicants had manuscripts posted to *bioRxiv*, which made evaluation of the work feasible for the local experts.

This strategy ultimately identified a slate of candidates for future consideration, most of whom were at the level of new or junior Assistant Professor, consistent with the majority of the applicant pool falling into this category. This group of candidates included 33% female individuals and 66% male individuals and 67% of candidates from groups historically excluded from and underrepresented in STEM fields as generally defined by the National Institutes of Health^23^. The focus here is on subsequent actions taken to evaluate and interview this pool of outstanding candidates at the Assistant Professor level.

### Zoom interview Phase 1: a set of common questions

Following evaluation of submitted applications, we invited a set of candidates at the level of Assistant Professor to participate in a 30-min Phase 1 Zoom interview. The Zoom interview included the trainee members of the search committee. Each candidate interviewed was provided with a slide illustrating the composition of the search committee, which included pictures, names, positions, and pronouns to illustrate the diversity of the committee and allow candidates to familiarize themselves with the search committee members. The trainees were provided with the application materials for each of these candidates with the exception of the confidential letters of recommendation. Each Zoom interview consisted of a standard set of questions (Box 1), with each question asked by the same member of the search committee for each candidate. The questions were agreed upon by the committee and included a question developed and asked by the trainees (Person #4 in Box 1). These meetings were held over a 2-day period when all search committee members were available. During the meeting, members of the search committee briefly introduced themselves and then proceeded through the set of questions, leaving time for the candidate to ask questions at the end. The Department Chair was present as an observer, primarily to answer any questions during this phase of the interview. Each candidate was briefly discussed after each interview with final decisions about who to invite for a more extended interview made after all zoom interviews were completed.

Box 1 Phase 1 Zoom Questions. For the first phase of the interview, all candidates were asked the same set of questions, allowing us to directly compare how candidates respond to a common set of questions **Zoom interview process (Phase 1)**

**Introduction of everyone in the virtual room (quick). (*2*** ***min total*)**

**Person #1**: Our goal here is to let you tell us what you are excited about and we are going to start with your research. As Biology faculty, we have the opportunity to advise a few first-year students each year. Imagine that you have a brand-new student in your office. They may be excited about biology or they may not know much. If you had a minute or two to explain your research to this student- how would you describe the work in your lab (idea is to get the elevator speech for this student)? (*3* *min*)

**Person #2**: We’ve read your research statement, and we have a few questions about how to position your research within the broader field.  What are you most proud of in terms of the research you have done, what do you see as the most exciting or innovative aspect of your future research? Why? Why are you the right person to do this work—what sets you apart from others? (*5* *min*)

**Person #3**: Why Emory and why the undergraduate Biology Department?  What have you heard about Emory and/or our department that would make it attractive to you?  (*3* *min*)

**Person #4** (Trainee): How do you envision engaging trainees, including graduate students and undergraduate trainees in your research program? How scalable is your work for engaging trainees at different levels?

How have you/would you tailor your mentorship approach to support trainees with different career goals, including those who don’t intend to stay in academia? (*5* *min*)

**Person #5**: What undergraduate courses would you be excited to teach? What new classes, at the graduate or undergraduate level, would you be excited to develop? (*3* *min*)

**Person #6**: Describe something you did that you are proud of to change a process or how you functioned as part of a program to advance diversity, equity, and inclusion at your current institution? How do you plan on engaging in DEI efforts at Emory? (3–5 min)

**Person** **#7**: Thanks for answering our questions—now we’d love to answer yours. (*5* *min*)

### Identifying candidates for further consideration

Following the Phase 1 Zoom interviews, we took several actions to identify the candidates that would move forward in the process. First, we had a meeting to discuss the Zoom interviews of each candidate and gather feedback from the entire committee based on our holistic review of: research, teaching, and actions toward DEI. Due to the very broad research base of these candidates, we reached out to colleagues at Emory to provide feedback on the proposed research programs, as well as the potential for interactions and collaborations with existing Emory faculty. With respect to teaching, the committee consisting of a graduate student, a post-doc, and both junior and senior members of the Biology Department felt well equipped to evaluate teaching among the candidates. Finally, we had the de-identified DEI statements of these candidates further evaluated by four colleagues using an updated rubric developed by the trainee members of the committee. With all these data in hand, all members of the committee were charged with ranking the candidates (1: Absolutely interview; 2: Yes interview; 3: Maybe Interview; 4: No interview). The trainees were asked to meet and agree upon their ranking, which was included with the rankings from the members of the search committee to identify a top group to invite for the next round of the interview. This group invited to participate in the Phase 2 interview consisted of 50% female individuals and 50% male individuals. Furthermore, 60% of the individuals invited for interviews are from groups historically underrepresented in/excluded from STEM fields.

### Prior to the interviews: preparing the landscape within the department

Before starting the faculty search process and engaging more broadly with candidates, we took several actions to prepare the department. First, a member of the department performed an anonymous survey of recent hires into our department regarding their own interview processes. This survey provided insights to some positive and negative experiences from the perspective of those who had recently been through the process. Second, we dedicated a department seminar to discuss the topic of implicit bias^24^, something we have done prior to the last several searches. This lively discussion each year reminds all of us that we have implicit biases that cannot be erased or eliminated. We also use this forum to remind the department community of best practices and behaviors when interacting with job candidates, such as not asking any candidate personal questions regardless of the intention. We also set ground rules for faculty candidate seminars and chalk talks to facilitate a robust (albeit virtual) discussion, while ensuring that each candidate is allotted sufficient time to complete their planned presentation.

### Zoom interview Phase 2: the interview process

Given the timing of these interviews, held while a global pandemic was raging, the Phase 2 interview, which would typically be an in person visit to campus, was conducted virtually. Significant time and effort was placed to arrive at a virtual Zoom schedule that would showcase the department without inducing mind-numbing Zoom fatigue for the candidate. In addition, we took actions to ensure an equitable process. We scheduled each interview for ~one and half days with one full day that would include the seminar and the chalk talk and one additional half day of meetings (Box 2).

Ahead of the interview, each candidate was assigned a faculty host who would provide details and answer any questions and was also connected to the department administrator who handles all logistics and scheduling. The faculty host reached out to the candidate with an e-mail that was common to all candidates providing information about the seminar, including the broad audience within the department, as well as the logistics of the “visit” and the chalk talk. Each candidate was asked for a list of faculty, both in the department and beyond, that they would like to meet. The schedule, which was assembled from the list of requests in consultation with the faculty host, was carefully balanced to fulfill the requests and limit time on Zoom. Beyond organizing the schedule, the administrator reached out to every candidate to offer any support needed to facilitate their interview process. The goal here was to create an equitable process for all candidates despite the fact that were interviewing from different places and potentially very different environments. Possible support could have included a hotel room for the duration of the interview, child or elder care, purchase of food, or anything else that would allow the candidate to have an optimal interview process and environment. At this time, the administrator also made inquiries about any scheduling requirements for the virtual interview that might be required such as the need to care for a child or address health or wellness needs. Only the administrator was part of the discussion and no member of the search committee or the faculty had any information about whether any candidate accepted this offer. This information was clearly communicated to the candidates to reassure them that taking advantage of this offer in any way would not be considered as part of the evaluation. The goal was to have an equitable process, which would also accommodate any individual needs of the candidates.

Each virtual interview started with a brief Zoom meeting the day before the interview with the Chair of the search committee and the faculty host. This meeting was held to go over the schedule, address any questions about the seminar/job talk or the chalk talk, and test the Zoom room. Day 1 of the interview process started with the job talk seminar. For the seminar, a host was assigned to field questions and monitor the chat. Having a host, who was always a senior member of the faculty in this role, allowed the seminar to stay on track with questions reined in to allow each candidate to finish their presentation. This strategy is important as data show that some speakers are more likely to be interrupted than others during a job talk^25^. Starting the day with the job talk is purposeful to both allow the candidate to get a major hurdle out of the way and to allow subsequent meetings to address any questions based on the seminar. The rest of the day proceeded with meetings with faculty. While these are traditionally one-on-one meetings, we opted for two faculty to meet with the candidate in most of these meetings. This allowed us to minimize Zoom time but still permitted many faculty to meet with the candidate. Recall that the virtual nature of this interview process removes multiple opportunities for informal interactions with the candidate, including typically at least two dinners and one breakfast. We carefully considered the dynamics when setting up the meetings with faculty so that the candidate would not be faced with two very senior faculty at once, for example.

The day included several breaks, including one for lunch. Following lunch, the candidate typically had a coffee break with trainees. This group consisted of undergraduate and graduate students as well as post-docs, typically in the general research area of the candidate. This meeting was not for the trainees who played a formal role in the search committee. The trainee members of the search committee met with each candidate in a formal meeting (the two trainees and the candidate). We then added another break before the chalk talk. Like the job talk seminar, the chalk talk was hosted by a senior member of the faculty. The virtual chalk talk was an event that instilled much creativity in the candidates. We allowed candidates to use any approach that they were comfortable with, including a few slides to outline their plans. After the chalk talk, the candidate was done for the day.

Day 2 started with coffee with the junior faculty. This meeting included as many junior faculty as possible to allow some informal/social interaction. Day 2 included a few meetings, followed by a final meeting with the Department Chair to address any questions. At some point during the interview process, the candidate also met with the Emory College Dean of the Faculty.

All of the Phase 2 interviews were conducted over a one-month period, generally hosting two candidates per week. This schedule aligns with how the department has generally conducted interviews, seeking to have a rapid timeline both to allow most direct consideration of different candidates and to complete the interview process in a timely fashion to accommodate the timelines of the applicants.

Box 2 Schedule for the Phase 2 virtual interview **Zoom interview process (Phase 2)**

**The night before**

- The Chair of the Search and the host of each candidate would hold a brief meeting to go over the schedule and answer any questions

**Day 1 (a full day)**

- Job talk seminar held as first event in the morning to allow the candidate to share their research prior to individual meetings
- Individual meetings that often included two faculty with consideration given to the composition of the group
- Meeting with trainee members of the search committee
- Breaks throughout the day, including for lunch
- Afternoon coffee break with trainees (not members of the search committee)
- Meeting with the Dean of the Faculty
- Break prior to the chalk talk to allow the candidate to prepare
- Chalk talk held at 4 p.m.–5 p.m. with a host who oversees the questions and monitors the chat

**Day 2 (limited to ~1/2 day)**

- Day 2 starts with an informal coffee hour with junior faculty
- Final meeting with Department Chair to address any final questions

### The offers: ranking the final candidates

After each interview, we solicited feedback using a Google form from all faculty and trainees that interacted with each candidate. We collated these data, which included rankings on a scale of 1–5 according to the same scale used previously: 1 = exceptional, 2 = excellent, 3 = average strong candidate, 4 = good, 5 = fair. We also included a straight yes/no question about support for an offer to each candidate. This feedback was then provided to the search committee, which convened to discuss each candidate. We then charged the search committee with ranking all candidates. As in prior evaluations, the trainees were asked to produce a consensus ranking. The trainees also provided feedback on each of the candidates. These recommendations and rankings, including the feedback from the broader community on ranking of 1–5 and the yes/no on an offer, were then shared at a faculty meeting to decide on final offers and to rank all candidates. The entire faculty was charged with some points to consider for their votes and ranking (Box 3). The entire faculty then voted on the plan and order of offers to be extended.

Box 3 Factors to consider for final ranking of the candidates

- **Factors TO consider in individually ranking candidates**:

- Evidence and/or potential for impact in their field (primarily) and within Biological Sciences more broadly (secondarily) with their research questions and trajectory
- Will they have colleagues here that they can connect to as they build their research program, in Biology (primarily) and beyond (secondarily)
- Evidence for and/or likelihood of being able to effectively teach and mentor students in relevant undergraduate majors and graduate programs that Biology faculty participate in
- Potential for mentoring trainees, including undergraduate and graduate students as well as post-doctoral fellows
- Evidence of and potential for impact with what a candidate brings to the table for diversity, inclusion and equity in our department, for their future trainees, and for Biology students.
- Furthermore, you can consider that some candidates fall below a bar for further consideration, and this is important information.
- **Factors NOT TO consider in individually ranking candidates:**
- Balancing the research fields of candidates being ranked
- Personal/career factors that the candidate’s situation poses (e.g., significant others, current position, etc.)
- Whether a candidate is a good fit for other departments at Emory

### The outcome: the offers

Ultimately, after much discussion and voting to rank individual candidates, the faculty narrowed the large pool of applicants and agreed four candidates that could be offered a position. Negotiation with the Dean’s office meant that three offers were made initially and when two of these candidates declined the offer, an additional offer was extended. In the end, we were fortunate to recruit two outstanding new colleagues to the department, so we consider this search an unmitigated success.

### Lessons learned: considerations for the future

The pandemic forced everyone to make drastic changes to virtually all aspects of their lives, including the search process described here. While all of us would prefer never to undertake a search under pandemic conditions again, the challenges have provided an opportunity to rethink how we conduct searches, in ways that may have positive outcomes for diversity even after society reaches a new normal. A number of the lessons learned align with a set of recent recommendations to streamline the search and interview process^26^.

With respect to the ad and the general search strategy, the broad search for a biologist was successful in generating a deep pool of highly qualified applicants (585 applications—60% male/37% female/3% Other or declined to answer and 13% underrepresented in STEM) with extremely diverse research foci. By way of comparison, a pre-pandemic search for a computational scientist generated a pool of only 16 applicants (94% male/2% female/<1% Other or declined to answer and no individuals from groups underrepresented in STEM), while a search at that time for an experimentalist in epigenetics/epigenomics brought in 97 complete applications (66% male/32% female/2% Other or declined to answer and 7% underrepresented in STEM). Overall, the percentage of individuals that diversify the pool of applicants was slightly improved in the current search compared to previous recent searches—likely because efforts to disseminate the ad widely were similar to efforts in previous searches. However, in any one year, one cannot know a priori from which subfields the most talented candidates will come, so a wider pool of total applicants naturally increases the raw number of viable applicants across identity categories that can help diversify the professoriate. Furthermore, research shows that when there are more individuals from various categories, evaluators can differentiate among them and rely less on group-based schemas, an effect that has been seen to increase how female applicants are rated^27,28^. An alternative approach is to specifically search in a research area that could enrich for individuals that diversify the STEM community as there is clear evidence of topic-specific bias^21^; however, this strategy may be challenging for a search process and may align better with specific recruitments. In a broad search, care must also be taken to avoid bias against specific research areas due to clear evidence that research in some areas is undervalued^21^. For the purposes of this search with a larger pool of total applicants than we typically see, the raw number of applicants in all categories, including those in groups typically underrepresented, increased significantly, achieving one of the major goals of this approach.

Evaluating a large number of applications is inherently challenging and the impact of implicit bias on such a large, time-consuming task is a major concern^29^. A potential strategy to overcome these challenges is the use of anonymized research statements as a first phase of the review process^30,31^. With the approach described here aimed at generating such a deep pool of applicants, an initial anonymized review process likely would not have been feasible. Faculty searches have historically been conducted in a largely traditional manner but without guidelines/consensus on the approach and/or ways implicit bias can be minimized. In this search, we sought to minimize bias by casting a wide net that would not allow for anonymized research statements. In contrast, if a department is looking for an individual to fill a particular niche, then such a wide search is unlikely to be fruitful and other ways to reduce bias with their smaller applicant pool need to be considered. In sum, searches should be adaptable and departments need to flexible if they are truly invested in decreasing bias and building a department increasing diversity, equity and inclusion are valued. These considerations show that different approaches can be employed in an attempt to combat bias in the initial phase of the review process^16^.

A point of common debate is when or even whether to request letters of recommendation. With 585 applicants requesting three letters each, this created a total of 1755 letters provided, arguably a colossal waste of valuable time^26^. Owing to a delayed approval for the search, we started the process late, so opted to include the request for letters with the initial application material. However, there is debate about the inherent value of such letters^26^, so either collecting them much later in the process, perhaps for those candidates in the final interview pool or even not at all, is a possibility to consider for the future.

Inclusion of trainees as formal members of the search committee was new for our process. This was one of the most successful modifications made and one that will be continued in the future^13^. A survey of department faculty revealed that only 38% of faculty were “extremely enthusiastic” about including trainees on the search committee prior to the search while that number increase to 65% following the search. The trainees were very engaged in the process and having met with each candidate, they could provide a fair comparison across all those interviewed. Points of debate included how much access to give trainees to confidential applicant material and how to value the critical input from these members of the search committee. While the trainees were not included in the final faculty vote deciding who should be offered a position, their opinions on each candidate were shared at this final discussion. Their thoughts on how they would feel in a classroom or laboratory setting with each faculty candidate were invaluable. The trainees both brought key insights to the process and benefited from their engagement in the process^13^.

A number of departments have employed a virtual interview (the Phase 1 Zoom interview described here) to make initial contact with candidates^26^, but this will be a new step for some. This approach has significant value for narrowing the number of candidates to move to the next phase of the process. Typically, both the answers to the questions about “why this position”, as well as the questions that the candidates pose can provide some measure of their interest in the position. As a Biology department within a college, gauging interest in teaching is critical to us and many applicants in the biomedical sciences remain uniformed about differences between positions within a medical school with minimal teaching and a college environment where teaching is a major mission. Thus, questions about teaching can be very informative in this scenario.

Faculty candidates often have partners who are also faculty candidates. This was true for several of our applicants. The ability to be flexible and aid in the identification of an additional position remains one of the most productive ways to ensure a successful hire. Turning the “two body problem” into a “two body opportunity” takes some time and effort^32^, but the outcome can often lead to an even more productive search than originally anticipated.

A major challenge was the potential to unintentionally inject inequity due to the inability to bring candidates on site for an interview process (Phase 2 Zoom interview described here) that typically consumes one or more days. When individuals are on site, they stay in the same hotel and, we have always presumed, have the same ability to focus on the events of the day(s). When candidates are in their own environment, there may be personal circumstances that impact their ability to focus on the interview. We attempted to address potential inequities by offering candidates financial support to help them optimize their local interview environment. As members of the search committee were blinded to whether candidates took advantage of this offer, we have no information about who accepted this offer and/or how these offers did or did not affect the process. Perhaps a consideration for the future would be an offer of financial support for childcare, elder care, etc. to allow all individuals the same opportunity to be absent from home and focused on the local activities.

Another challenge of moving the typical 2-day, in person interview to a virtual format was balancing time to interview the candidate and introduce the candidate to the department with the reality of Zoom fatigue^33^. For this reason, we decided to limit any social events to a coffee hour with the junior faculty. We also built in several breaks throughout the day and had the administrator consult with each applicant to identify any specific needs they might have regarding the scheduling. Ultimately, having this phase of the interview process in person is far preferable to navigating the virtual process.

### Summary and outlook

A successful search takes a great deal of time and effort dedicated from the search committee, as well as all members of the department. The input from trainees, which was new to our process, was invaluable. We will seek to incorporate some of the lessons learned during this recent experience in future searches, with the caveat that we all hope to hold future searches in person. However, there are aspects of the virtual process that may enhance equity and, thus, should be considered for the future. Each search is unique with distinct challenges and opportunities, but there are concrete actions we can take to mitigate bias in the process.

## Supplementary information

Supplementary Information

## References

[1] Tilghman S (2021). Concrete steps to diversify the scientific workforce. Science (N. Y., N. Y.).

[2] Olzmann JA (2020). Diversity through equity and inclusion: the responsibility belongs to all of us. Mol. Biol. Cell.

[3] Uhlmann EL, Cohen GL (2007). “I think it, therefore it’s true”: effects of self-perceived objectivity on hiring discrimination. Organ. Behav. Hum. Decis. Process..

[4] Moss-Racusin CA, Dovidio JF, Brescoll VL, Graham MJ, Handelsman J (2012). Science faculty’s subtle gender biases favor male students. Proc. Natl. Acad. Sci. USA.

[5] Rivera LA (2017). When two bodies are (not) a problem: gender and relationship status discrimination in academic hiring. Am. Sociological Rev..

[6] White-Lewis DK (2020). The facade of fit in faculty search processes. J. High. Educ..

[7] Gibbs KD, Basson J, Xierali IM, Broniatowski DA (2016). Decoupling of the minority PhD talent pool and assistant professor hiring in medical school basic science departments in the US. eLife.

[8] Gómez-Van Cortright, G. In *ASBMBToday* (American Society for Biochemistry and Molecular Biology, 2021).

[9] Mohr, T. S. In *Harvard Buisiness Review* (Harvard Business Publishing: Higher Education, 2014).

[10] Callier, V. In *Scientific American* (Springer Nature, 2016).

[11] Gibbs KD, McGready J, Bennett JC, Griffin K (2014). Biomedical science Ph.D. career interest patterns by race/ethnicity and gender. PLoS ONE.

[12] Hakkola, L. & Dyer, S. J. V. Role conflict: How search committee chairs negotiate faculty status, diversity, and equity in faculty searches. *J. Divers. High. Educ.*10.1037/dhe0000386 (2022).

[13] Thomas, K. & Nguyen, K. A model for diversifying faculty recruitment. *Nature*, 10.1038/d41586-021-02726-w (2021). 10.1038/d41586-021-02726-w 34611346

[14] Cahn, P. S., Gona, C. M., Naidoo, K. & Truong, K. A. Disrupting bias without trainings: the effect of equity advocates on faculty search committees. *Innov. High. Educ*. 10.1007/s10755-021-09575-5 (2021). 10.1007/s10755-021-09575-5 PMC8383923 34456457

[15] Sylvester, C.-Y. C., Sánchez-Parkinson, L., Yettaw, M. & Chavous, T. The promise of diversity statements: insights and an initial framework developed from a faculty search process. *Curr.**J. Divers. Scholar. Social Change* **1**, 153–169 (2019).

[16] Fernandes JD (2020). A survey-based analysis of the academic job market. eLife.

[17] Fu, D. Y. & Hughey, J. J. Releasing a preprint is associated with more attention and citations for the peer-reviewed article. *Elife* **8**, 10.7554/eLife.52646 (2019). 10.7554/eLife.52646 PMC6914335 31808742

[18] Sever, R. et al. bioRxiv: the preprint server for biology. Preprint at *bioRxiv* 833400, 10.1101/833400 (2019).

[19] Day AE, Corbett P, Boyle J (2020). Is there a gender gap in chemical sciences scholarly communication?. Chem. Sci..

[20] Pickett CL (2019). The increasing importance of fellowships and career development awards in the careers of early-stage biomedical academic researchers. PLoS ONE.

[21] Hoppe TA (2019). Topic choice contributes to the lower rate of NIH awards to African-American/black scientists. Sci. Adv..

[22] Erosheva EA (2020). NIH peer review: Criterion scores completely account for racial disparities in overall impact scores. Sci. Adv..

[23] Valantine HA (2020). NIH’s scientific approach to inclusive excellence. Faseb J..

[24] Pritlove C, Juando-Prats C, Ala-Leppilampi K, Parsons JA (2019). The good, the bad, and the ugly of implicit bias. Lancet.

[25] Blair-Loy M (2017). Gender in engineering departments: are there gender differences in interruptions of academic job talks?. Soc. Sci..

[26] Golde, C. M. 5 Easy fixes for a broken faculty job market. *Chronicle of Higher Education* (September 6th, 2019).

[27] Sackett PR, DuBois CL, Noe AW (1991). Tokenism in performance evaluation: the effects of work group representation on male-female and White-Black differences in performance ratings. J. Appl. Psychol..

[28] Heilman ME (1980). The impact of situational factors on personnel decisions concerning women: varying the sex composition of the applicant pool. Organ. Behav. Hum. Perform..

[29] Russell J, Brock S, Rudisill M (2019). Recognizing the impact of bias in faculty recruitment, retention, and advancement processes. Kinesiol. Rev..

[30] ÅSlund O, Skans ON (2012). Do anonymous job application procedures level the playing field?. Ind. Labor Relat. Rev..

[31] Langin, K. In *Nature Careers*10.1126/science.caredit.abl7645 (Nature, 2021).

[32] Gagliano Taliun, S. A. One scientist couple’s five suggestions to solve the ‘two body problem’. *Nature*10.1038/d41586-021-00917-z (2021). 10.1038/d41586-021-00917-z 33824533

[33] Wiederhold BK (2020). Connecting through technology during the coronavirus disease 2019 pandemic: avoiding “zoom fatigue”. Cyberpsychol. Behav. Soc. Netw..

