# Supplementary material for: Proactive strategies for an inclusive faculty search process
Source: Commun Biol. 2022 Jun 16;5:592. doi: 10.1038/s42003-022-03545-z (PMC9203751; doi:10.1038/s42003-022-03545-z)
Supplement: Supplementary file 1 — Supplementary Information [file 42003_2022_3545_MOESM1_ESM.pdf]

Supplementary Note 1: Rubric to Assess Diversity, Equity, and Inclusion (DEI) Statements

Please note, this rubric is not meant to be a prescriptive tool. To achieve the best outcomes, we recommend that committees using this rubric should preemptively discuss the specific needs of their department and calibrate the rubric accordingly. During the assessment process, reviewers should use the rubric score *and* discuss the content of the DEI statement to holistically assess each candidate (i.e., the rubric score alone should not determine a candidate's ranking).

**Problematic Approaches**	
**0**	*Solely* focuses on how their teaching, research, and/or service could theoretically address DEI, but the ideas are not entirely novel, feasible, practical, or impactful
	Solely focuses on their belonging to an underrepresented group as evidence they completely understand barriers other individuals could face in the academy
	Solely acknowledges that racism, classism, etc. are issues in the academy
	Solely discusses how they are knowledgeable about DEI through passive participation in a few workshops, sessions, reading groups, etc.
	Solely provides personal anecdotes about how they have been discriminated against or have discriminated against someone as evidence they value and understand DEI

**Valuing and Understanding Diversity, Equity, and Inclusion**	
**1-2**	Has given little to no effort on increasing their knowledge/understanding of DEI-related topics through workshops, communication, etc.
	Simply discusses DEI in vague terms and does not describe how they would work to improve DEI in their lab/classroom/department/university/community
**3**	Recognizes and places significance on their role as a faculty member in shaping and supporting DEI efforts in their lab/classroom/department/university/community
	Expresses willingness to discuss and confront challenges related to advancing DEI practices with the broader community of undergraduates, graduate students, postdoctoral fellows, faculty, and staff
**4-5**	Demonstrates an understanding that diversity has many dimensions (e.g., ethnic, socioeconomic, racial, gender, sexual orientation, disability, cultural differences, etc.) and that individuals have unique experiences given their intersectionality along these dimensions
	Vocalizes that antiracism practices requires consistent and long-term growth, reflection, and engagement (and that they are prepared to put in this work)

**Track Record in Advancing Diversity, Equity, and Inclusion**<br>Note: For this section, keep in mind that individuals may come from departments that were hostile to DEI-related activities, so they may not have felt comfortable participating.	
**1-2**	Has invested little time in advancing DEI beyond basic expectations for their academic rank or institutional climate
	Passively describes past participation in workshops, committees, etc. and does not describe the purpose, outcome, or their specific role in such events or organizations
**3**	Shows limited participation in single activity but provides a clearer description of the objectives/results of activity and/or the individual role they played

**4-5**	Demonstrates strong leadership role in past groups/projects that support underrepresented students at various levels (e.g., undergraduate, graduate, postdoctoral)
	Leadership in DEI extends to organizing events aimed at the departmental level to increase representation and better support underrepresented students as well as colleagues
	Documents continuous participation in events or organizations geared towards advancing DEI (can include work completed outside of academia, e.g., community activism) during multiple career stages

Track Record in Mentoring Diverse Trainees

Note: For this section, keep in mind the difference between diverse and BIPOC (black, indigenous, and people of color). I.e., a white male who mentors a white woman 10 years their senior can honestly say they mentored a diverse trainee however has no interaction with trainees from historically underrepresented groups in STEM and therefore cannot advocate or be an ally for these trainees.

**1-2**	Briefly mentions inclusion in curriculum, but has no plans to implement additional teaching strategies that enhance inclusion
	Shows little evidence of personal actions taken to mentor diverse students in the classroom or the lab (e.g., I had a diverse classroom and they did fine)
**3**	Has taken a few workshops dedicated to enhancing intercultural or intergroup competencies and skills
**4-5**	Demonstrates evidence (through specific strategies) of how they have updated their syllabus, teaching approach, course curriculum, etc. to enhance representation and retention of underrepresented groups
	Demonstrates engagement in long-term mentorship program(s) that supports underrepresented groups
	Identifies continued commitment towards evaluating and assessing inclusive teaching practices and offers suggestions on how

Plans for Advancing Diversity, Equity, and Inclusion

**1-2**	Does not verbalize a plan for advancing DEI beyond general expectations for all faculty as outlined by the department (no personal agency or motivation)
	Describes a vague plan for how they will create an inclusive classroom or lab space without clear actionable items that they intend to accomplish to reach that goal. The plan lacks detail/purpose (e.g., if "outreach" is proposed, there is no mention of the specific target, the type of engagement, or expected outcomes)
**3**	Mentions plans or ideas they intend to implement to advance DEI and provides clear and detailed ideas for what existing programs they would get involved with (with reference to current activities/limitations) as appropriate for their academic rank
**4-5**	Presents ways in which their research, teaching, and/or service will advance DEI in the university, their academic societies, or the broader community
	Addresses multiple areas of need (e.g., classroom climate, the laboratory, conferences)
	Presents clear way of evaluating plans along with their impact. May also describe the growth of their plan over time

This rubric has been adapted by Kyle A. Thomas and Karena H. Nguyen. K.A.T is graduate student in the Biomedical Engineering Department at Emory University and the Georgia Institute of Technology. K.H.N. is a postdoctoral fellow in the Biology Department at Emory University. Sources include Berkeley's Rubric for Assessing Candidate Contributions to Diversity, Equity, and Inclusion, University of Michigan Center for Research on Learning and Teaching (CRLT) and Rackham Graduate School's Diversity Statement Evaluation Rubric, 5 Don'ts in Writing Your DEI Statement, and Sylvester et al. 2019 (DOI: http://dx.doi.org/10.3998/currents.17387731.0001.112).

VERSION 2021-09-27
